# Enhancing capacity for risk factor surveillance at the regional/local level: a follow-up review of the findings of the Canadian Think Tank Forum after 4 years

**DOI:** 10.1186/2049-3258-72-2

**Published:** 2014-01-22

**Authors:** Bernard CK Choi, Mary Lou Decou, Drona Rasali, Patricia J Martens, Michelina Mancuso, Ronald C Plotnikoff, Cory Neudorf, Joanne Thanos, Lawrence W Svenson, Keith Denny, Heather Orpana, Paula Stewart, Michael King, Jane Griffith, Tannis Erickson, Renate van Dorp, Deanna White, Amira Ali

**Affiliations:** 1 Centre for Chronic Disease Prevention, Public Health Agency of Canada (PHAC), Government of Canada, Ottawa, Ontario K1A 0 K9, Canada; 2 Dalla Lana School of Public Health, University of Toronto, Toronto, Ontario, Canada; 3 Department of Epidemiology and Community Medicine, University of Ottawa, Ottawa, Ontario, Canada; 4 Population Health Surveillance & Epidemiology, British Columbia Provincial Health Services Authority, Vancouver, BC, Canada; 5 Faculty of Kinesiology & Health Studies, University of Regina, Regina, Saskatchewan, Canada; 6 Manitoba Centre for Health Policy; Department of Community Health Sciences, Faculty of Medicine, University of Manitoba, Winnipeg, Manitoba, Canada; 7 Performance Measurement, New Brunswick Health Council, Moncton, NB, Canada; 8 Physical Activity and Population Health Education; Priority Research Centre in Physical Activity and Nutrition, The University of Newcastle, Callaghan, NSW, Australia; 9 Department of Community Health & Epidemiology, College of Medicine, University of Saskatchewan, Saskatoon, Saskatchewan, Canada; 10 Saskatoon Health Region, Saskatoon, Saskatchewan, Canada; 11 Public Health Planning and Liaison Branch, Public Health Division, Ministry of Health and Long-term Care, Toronto, Ontario, Canada; 12 Epidemiology and Surveillance, Surveillance and Assessment Branch, Alberta Health, Edmonton, Alberta, Canada; 13 Department of Community Health Sciences, University of Calgary, Calgary, Alberta, Canada; 14 Department of Public Health Sciences, School of Public Health, University of Alberta, Edmonton, Alberta, Canada; 15 Canadian Institute for Health Information, Ottawa, Ontario, Canada; 16 Population Health Promotion and Innovation Division, Centre for Health Promotion, Public Health Agency of Canada (PHAC), Government of Canada, Ottawa, Ontario, Canada; 17 School of Psychology, University of Ottawa, Ottawa, Ontario, Canada; 18 Medical Officer of Health, Leeds Grenville and Lanark District Health Unit, Brockville, Ontario, Canada; 19 Sudbury and District Health Unit, Rapid Risk Factor Surveillance System (RRFSS) Steering Group, Sudbury, Ontario, Canada; 20 Epidemiology and Cancer Registry, Cancer Care Manitoba, Winnipeg, Manitoba, Canada; 21 Perth District Health Unit, Stratford, Ontario, Canada; 22 Haldimand-Norfolk Health Unit, Simcoe, Ontario, Canada; 23 Ottawa Public Health, Ottawa, Ontario, Canada

**Keywords:** Public health surveillance, Capacity building, Behavioural risk factors

## Abstract

**Background:**

National health surveys are sometimes used to provide estimates on risk factors for policy and program development at the regional/local level. However, as regional/local needs may differ from national ones, an important question is how to also enhance capacity for risk factor surveillance regionally/locally.

**Methods:**

A Think Tank Forum was convened in Canada to discuss the needs, characteristics, coordination, tools and next steps to build capacity for regional/local risk factor surveillance. A series of follow up activities to review the relevant issues pertaining to needs, characteristics and capacity of risk factor surveillance were conducted.

**Results:**

Results confirmed the need for a regional/local risk factor surveillance system that is flexible, timely, of good quality, having a communication plan, and responsive to local needs. It is important to conduct an environmental scan and a gap analysis, to develop a common vision, to build central and local coordination and leadership, to build on existing tools and resources, and to use innovation.

**Conclusions:**

Findings of the Think Tank Forum are important for building surveillance capacity at the local/county level, both in Canada and globally. This paper provides a follow-up review of the findings based on progress over the last 4 years.

## Background

In Canada, public health program planning and delivery at the regional/local level is performed by the regional health authorities (RHA). Major national health surveys, such as the National Population Health Survey (NPHS) [1], Canadian Community Health Survey (CCHS) [2], and Canadian Health Measures Survey (CHMS) [3], provide important reliable national statistics on chronic diseases and their risk factors for public health planning. However, national health surveys cannot always address regional/local needs, usually have a limited number of questions, and are sometimes considered neither timely nor frequent enough [4]. Some national surveys may not have sufficient sample sizes to address the needs of smaller geographies.

There are pockets of risk factor surveillance activities at the provincial/territorial and regional/local level to collect additional data from local surveys and administrative databases to provide sub-national estimates across Canada (Appendix). For example, in Saskatoon Health Region, supplementary health surveys are conducted periodically for purposes of public health surveillance, research and needs assessment, and reported to the community and to decision makers [5]. In Ontario, the Rapid Risk Factor Surveillance System (RRFSS) [6] was set up because some local health units felt the need to have a rapid and flexible information system to supplement data from national health surveys [4]. In British Columbia, a province-wide randomized telephone health and wellness survey (BC-HWS) was conducted to monitor the health behavior risk factors and general health at the local level [7].

In 2005, a federal/provincial/territorial report on "Enhancing Capacity for Surveillance of Chronic Disease Risk Factors and Determinants" recommended to "establish locally/regionally coordinated ongoing flexible public health data collection systems (such as the Rapid Risk Factor Surveillance System in Ontario)" [8]. The purpose of regional/local data is to "expand data sources to fill gaps in surveillance knowledge". In addition, the task group discussed the use of both a "roll-down" approach from national to local level (e.g. CCHS may use sufficient sample sizes to provide local area estimates), and a "roll-up" approach from local to national level (e.g. local surveys may together provide estimates at the national level).

In order to further explore ways to enhance the capacity in collaborative regional/local level chronic disease risk factor surveillance, a Think Tank Forum was organized in Canada in 2008 which provided important insights and guidance on the issues relating to enhancing capacity for risk factor surveillance at the regional/local level. The Forum also established the Canadian Alliance for Regional Risk Factor Surveillance (CARRFS) [9] which has been in operation in Canada for four years. The objective of this paper is to report the findings of the Think Tank Forum, and to discuss progress made under each of the areas of recommendations. This is of importance for the operations of the CARRFS, and experts in other countries interested in building surveillance capacity at the local or county level.

## Methods

Invitations to participate in the Think Tank Forum were sent through professional contacts of the planning committee, the Public Health Agency of Canada (PHAC, including its regional offices), regional health authorities (including local health units and regional health departments), and the PHAC’s Chronic Disease and Injury Prevention and Control Expert Group whose members represent all provinces and territories in Canada. A total of 156 invitations were sent. Ethical approval was not required as the Think Tank Forum did not carry out experimental research on humans or animals.

The Think Tank Forum was held in Toronto, Ontario in February 2008. The objective was to invite as many experts as possible from all provinces and territories, who were working on or interested in risk factor surveillance at the local area level, in order to discuss how to support and build capacity for collaborative regional/local area chronic disease risk factor surveillance across Canada. The Think Tank was a one-and-a-half day forum consisting of an initial series of talks by plenary speakers followed by three small group discussions. Small group sessions were organized to respond to a series of questions which developed based on a standard approach to community development:

Q1. What are the needs for regional/local area surveillance?

Q2. What are the characteristics of regional/local area surveillance?

Q3. What are the needs to coordinate regional/local area surveillance?

Q4. What tools can support and build capacity for regional/local area surveillance?

Q5. What are the next steps to build capacity for regional/local area surveillance?

The first small group discussion session responded to Q1 and Q2; the second session responded to Q3 and Q4; and the third session responded to Q5. The first two discussion sessions each had four breakout groups, with randomly pre-allocated members to maximize interaction of members of different backgrounds and geographic locales. To maximize the amount of suggestions on future steps (Q5), the third session had 12 breakout groups. Suggestions from each session and group were recorded and later interpreted and synthesised by a working group comprised of authors for this paper.

## Results

A total of 108 chronic disease risk factor surveillance experts participated in the Think Tank Forum, representing federal (20 participants), provincial (29) and territorial (1) governments and agencies, local/regional health authorities (43), universities (5), non-government organizations (8), and international agencies (2). The forum started with invited plenary speakers providing local, provincial/territorial and national perspectives, as well as international perspectives from Pan American Health Organization (PAHO) and US Centers for Disease Control and Prevention (CDC) on chronic disease risk factor surveillance. The plenary speakers presented interesting viewpoints and background information to stimulate the thinking of the participants and to prepare them for the small group discussion sessions.

The first small group discussion session on "needs and characteristics of collaborative regional/local area risk factor surveillance" (Questions 1 and 2). The needs identified most frequently were: local data for local action, program development and evaluation, and special local data needs. The characteristics of regional/local area surveillance most frequently identified were: data flexibility, timeliness, quality, relevance and built-in knowledge translation systems. An extended list of answers to these questions is available from the authors.

### The need for regional/local area risk factor surveillance

In Canada, health surveys are conducted at three distinct levels - national, provincial/territorial, and regional/local. The Canadian Community Health Survey (CCHS) and Canadian Health Measures Survey (CHMS) are examples at the national level.

The next level is provincial/territorial surveys. For example, in Alberta, there is the provincial Alberta Health and Wellness (AHW) survey program to address gaps in the national health surveys and to be responsive to provincial needs (Appendix). The provincial surveys fill gaps from the national surveys, as they explicitly limit overlap in questions and are responsive to provincial needs. They are also more flexible and easier to change direction. Finally, regional/local surveys also exist. Examples include Saskatoon Health Region surveys in Saskatchewan [5] and the Rapid Risk Factor Surveillance System (RRFSS) in Ontario (Appendix) [6]. These continue to fill the gaps left by the national and provincial surveys and are more responsive to local requirements.

National health surveys, especially the CCHS with a large sample size of approximately 65,000 a year that can be pooled over time, can provide certain information at the provincial/territorial and regional/local levels. Provincial surveys can also provide information at the regional/local level. There are, however, important challenges to using large scale ("top down") surveys to generate local information.

First, national and provincial health surveys may not always meet the needs at the regional/local level, as local needs may differ from provincial and national needs. They do not allow targeted local questions, such as "Before this phone call had you heard of our Regional Health Department?" or "Have you seen or heard our smoke free home campaign?" They are not very flexible, as once the questionnaire consultation period is over, it is difficult to add or change questions. They are not timely enough. Regional and local health authorities need rapid data to respond to the emerging issues, but national surveys typically have a long lag time from completion of survey to release of results.

Second, there are sample size issues. National and provincial surveys may have insufficient sample size at the local level. While their sample sizes are determined to provide stable estimates of common diseases and risk factors at the health region level, sample sizes are often too small to allow for subgroup analysis within regions. To overcome sample size problems, the Canadian Institute for Health Information (CIHI) has worked with Statistics Canada to analyse the CCHS at regional (local health units) and sub-regional (Census Metropolitan Areas, CMA) levels. Even when two cycles of CCHS were combined, many of the estimates were suppressed for a number of the regions and CMAs due to small sample sizes. Researchers at the Manitoba Centre for Health Policy also found similar issues [10]. The CCHS model has moved from collection of a sample of 130,000 every two years to ongoing collection of 65,000 per year which should address some of this challenge by allowing for pooling of data. However, this may not allow for tracking changes between waves of the survey.

The need for regional/local area surveys exists. However, the format and method to roll up data from local to provincial to national levels will need to be established. By providing a forum for regional/local area surveillance leaders to connect, CARRFS has provided an important forum to facilitate sharing information to work towards solutions to this challenge.

### Characteristics of regional/local area surveillance

Ideal characteristics of surveillance at the regional/local level identified at the Think Tank Forum include: flexibility, timeliness, quality, communication plan, and responsiveness to local needs. While the ideal characteristics are nice to achieve, there are sometimes practical constraints. For example, due to resource limitations, surveys conducted at the regional/local level may not have the same degree of design and data strength as national surveys.

The Ontario RRFSS provides an example of the ideal characteristics versus the practical difficulties [6]. Created in 2000 after a pilot project in the Durham health region, RRFSS is the longest ongoing regional/local level risk factor survey system in Canada. Based on the initial results of the Durham pilot, a vision of a rapid, flexible, cost effective, survey-based surveillance system was proposed. It was thought that to achieve the ideal characteristics of timeliness, flexibility, and cost effectiveness, RRFSS should be based on a franchise model, a turnkey package, and a global support system [4]. The franchise model refers to a system where health regions can buy into a ready-made surveillance program to be implemented in their jurisdiction. It would comprise of turnkey package in that content would be developed centrally, and health regions would be able to choose from a ready-made menu of surveillance content. Finally, a global support system would comprise a centralised help desk and web site to provide access to statistical advisors.

After 14 years of operation, however, some of the initial performance indicators set forth in 2000 have been modified. Initially a monthly sampling frame was used, allowing data to be made available at the end of each month (timeliness), and permitting monthly changes in the questions (flexibility). This system worked well for eight years. However, in an effort to mitigate falling survey response rates, RRFSS switched to a four month sampling frame in 2009 – providing more time to contact selected households, and for refusal conversion. Data are therefore now made available every four months with a two month time lag (e.g., data collected from January to April are released in July). This is also the frequency at which changes to the questions can be made. These changes have contributed to a loss of timeliness, though flexibility and responsiveness to local needs are still being maintained overall. Also, questionnaire module development can take up to six months, which is not rapid.

Continued collaborative efforts are needed to work out ways to promote the ideal characteristics of regional/local area surveillance that are efficient and practical. In order to support this work at the regional/local level, CARRFS’ tools and resources working group is compiling a database of chronic disease risk factor surveillance tools and resources, which should facilitate the timely development of content for regional/local surveillance systems.

### The need to coordinate regional/local area surveillance

Following the 2008 Think Tank, an environmental scan was conducted by CARFFS in 2010 to provide a preliminary inventory of local risk factor surveillance activities being undertaken across Canada. The results of this scan have been reported elsewhere [11], and indicate that public health capacity at the regional/local level is uneven and in general could be strengthened. Coordination of regional/local surveillance efforts across the country, and pooling of resources and expertise, can increase capacity.

The second small group discussion session on "building collaborative regional/local area risk factor surveillance" (Question 4) discussed the needs to coordinate regional/local area surveillance and tools to support/build capacity for regional/local area surveillance. The themes identified most frequently for the need to coordinate regional/local area surveillance included: creating an inventory and conducting a gap analysis, visioning, and effective communication. Finally, responses to "What tools can support and build capacity for regional/local are surveillance" (Question 5) included filling gaps, maximizing information gain through innovation, and creating leadership capacity at all levels.

### Supporting collaborative regional/local area surveillance

Existing resources such as survey instruments and questions from national and provincial health surveys can be excellent tools to support regional/local health surveys. For example, questions from the national CCHS and NPHS have been adapted for use by health regions. CARRFS has struck a tools and resources working group in order to consolidate and share content that can be used by regional/local surveillance professionals.

Universities can play a role in developing new resources and tools to help regional/local health surveys. The Colchester East Hants Health Authority partnered with Dalhousie University, which produced a substantial dataset showing the rates of diagnosis and health system contacts for a number of conditions at the Community Health Board and finer level to inform Primary Health Care planning (Appendix). The Manitoba Centre for Health Policy of the University of Manitoba prepared reports to address regional health authority needs for chronic disease estimates on trends [12,13] and methodologies to measure chronic disease at the local level [14]. "*The Need to Know* Team" funded by the Canadian Institutes of Health Research (CIHR) is a research collaboration amongst university research scientists, RHA top-level planners, and Manitoba Health planners, to produce research for decision-making at the regional level [15,16].

Tools are needed to address the cost-effectiveness of using already-existing data, and the ability to track local populations and produce estimates at one point in time or longitudinally at very small geographical area levels. CARRFS has organized a number of learning events related to use of existing data and geographic information systems to meet this need.

The third small group discussion session on "making it happen" discussed Question 5 "What are the next steps to build capacity for regional/local area surveillance?" In Table 1, the suggested next steps are listed by group, as well as under the seven themes of capacity building [17]. The seven themes are grouped under the acronym "SCIENCE": *S*trategy, *C*ollaboration, *I*nformation, *E*ducation, *N*ovelty, *C*ommunication and *E*valuation.

**Table 1:** Results of small group discussion on "Q5: what are the next steps to build capacity for regional/local area surveillance?", by themes (7 themes) and by groups (12 groups), think tank forum, Toronto, Canada, 2008

**Theme/sub-theme**	**Next Steps**
1. Strategy	
1.1. Marketing strategy	• Promote risk factor awareness and knowledge to policy makers, public (groups 1 and 10).
	• Market local surveillance to local and regional decision makers (group 1).
	• Create a business case including a marketing plan - where do we need to go? (5 and 11).
	• Elevate the importance of chronic disease surveillance and raise profile of the work (5).
1.2. Legislation	• Develop legislation to mandate local risk factor surveillance at a minimum level, to be similar across the board (1 and 5).
	• Look at altering legislation regarding information held by Statistics Canada to increase ready availability of data (10).
1.3. National leadership	• Federal government (e.g. PHAC) leads, coordinates and facilitates, but not control (1).
	• Strong national leadership and mandate required from federal level to set the vision, set quality assurance standards, conduct validity research, provide national clearing house or resource centre for queries, and maintain consistency across provinces (2, 3, 6 and 9).
	• Provide flexible leadership to reflect changing needs of the provinces and territories (6).
	• At the national level, one must recognize the importance of local/small area data, provide technical support, mitigate redundancy and duplication, provide grants to help build capacity for some areas to create parity, and create a transparent format for application for funding (8).
	• Support at the national level for multi-modes, assessment, analysis and tools (9).
	• Make creation of the collaborative local area surveillance system a priority for PHAC (10).
	• PHAC coordinates in early stages but then this leads to a self-organization system (12).
1.4. Provincial/territorial leadership	• Strong provincial and territorial leadership and mandate required to coordinate local/regional groups, ensure balanced regional stakeholder representation, ensure equity of resources and capacity across the regions, enforce quality standards, tie together databases, and mediate relationships between database holders and research at universities (2, 3 and 6).
	• Province must work on resolving comparability issues and coordinate regional needs (8).
	• Provincial leadership in local area surveillance (e.g. provide common analysis resources, ensure consistent regular reports across local areas, roll out existing surveys to all health units and ensure methodology is appropriate for all local areas (9 and 10).
1.5. Regional/local leadership	• Regional and local leadership to define information needs, including core and additional data (3).
	• Need to gain understanding of local needs in order to avoid a "one size fits all" solution (4 and 8).
	• Establish local networks of surveillance (4).
	• Regional and local leadership to provide resources to implement the local system, engage policy makers (6).
1.6. Funding strategy	• Strategy to share human resources, expertise and funding between all federal/provincial/territorial and local levels (2 and 10).
	• Sustainable funding (7).
	• Identify tools for funding, find any potential pools of resources (8).
1.7. Clear vision	• Clear vision of the health goals and ultimate functions (3 and 5).
	• Clearly defined roles and responsibilities of national, provincial and local systems (3, 4 and 8).
	• Create a business plan and roadmap for surveillance activities (5 and 8).
	• Define common vision, including a common glossary of terms (11).
1.8. Network of networks	• Establish a country-wide network of local area surveillance networks across Canada (12).
	• Establish connections and deal with confidentiality and workflow issues (12).
2. Collaboration	
2.1. Identification of stakeholders	• Identify the stakeholders and ensure that the right people are at the table (1, 5 and 7).
	• Ensure that non-public health organizations (education, wellness, etc.) are brought to the table (3 and 9).
	• Engage policy makers, decision makers, clinical experts, members of other sectors and stakeholders (6).
	• Inclusiveness needs to be ensured including representation from all provinces and territories, and non-government groups (11).
2.2. Collaboration at federal, provincial/territorial, regional/local levels	• Support meetings of stakeholders at all levels (1).
	• Infrastructure (7).
2.3. Inter-sectoral working grops	• Create inter-sectoral working groups (1).
	• Support regional working groups including communities, programs, experts, education, health, NGOs, etc. (1).
	• Ensure inclusive and balanced representation in working groups (s 1, 5, 6 and 7)
3. Information	
3.1. Data sharing agreements	• Implement data sharing agreements (local data collection and ownership, but shared data for centralized analysis) (1 and 3).
	• Improve access to CCHS data and statistical expertise (4).
	• Augment CCHS, with age standardized rates and logistic regression (5).
	• Expand CCHS to build on what is happening nationally (e.g. oversample in certain health regions to collect local data) (8).
	• Explore existing non-health sources of health data (e.g. drivers’ licenses, taxes, census, passports, health cards, etc.) and remove barriers to accessing these data (10).
3.2. Data sharing practices	• Centralized repository (secure) with mapping and comparability features (2).
	• Sharing of GIS mapping to expand into local area data (3).
	• Data to be collected locally in all areas, to roll up to provincial estimates (4).
	• Create network architecture (mapping diagram and data pieces) (5).
	• Create and manage clearinghouse of what’s being done, and support information sharing (8).
3.3. Data standard	• Coordinate the creation of data standards (2).
	• Work must be mandated and funded to a minimum level for parity across the country (3 and 11).
	• Network should feature central analytic capacity and address inequities in localities and regions (4).
	• Centralized support (e.g. public health observatory) would help greatly with capacity and contribute to methods, core content, analysis and standard core content, and data sharing (5).
	• Conduct international benchmarking (7 and 11).
3.4. Standard questions	• Set up validated standard questions that meet local needs but allow national perspective (2).
	• Need a clear list of indicators with clear identification of key risk factors (5).
	• Consistent definitions of risk factors (7).
	• Establish a resource library which includes questionnaires and methods (10).
	• Identify what questions really need to be asked (12).
3.5. Reporting standard	• Provide standard templates for reporting (2).
3.6. Information dissemination	• Help disseminate results (2).
	• Interface for knowledge sharing (7).
	• All survey information is readily available and easily accessed (9).
3.7. Capacity mapping/information gap	• Perform capacity mapping for databases and competencies (3 and 6).
	• Conduct an inventory of what is currently being done, what the complementary network components are, and a gap analysis with focused consideration of special groups (e.g. children, First Nations, etc.) (5).
	• Conduct system inventory (6).
	• Conduct environmental scan of existing tools, systems and datasets (7 and 12).
	• Stay conscious of the needs of unique populations (7).
	• Perform a capacity assessment and environmental scan, and a gap analysis (11).
4. Education	
4.1. Training workshops	• Provide workshops and ongoing training (2 and 7).
	• Build capacity in data analysis (4).
	• Capitalize on existing resources including data and knowledge (12).
4.2. Newsletter	• Electronic newsletter to disseminate knowledge on new techniques and tools (9).
4.3. Public education	• Educate public that participation in surveys helps in local area planning, and which surveys are currently being done (10).
	• Link to and have presence at conference to build appetite and spread the word (10).
5. Novelty	
5.1. New methodology	• Foster research and development (R&D) to develop new surveillance methodologies, and a better understanding of what is feasible in different geographic areas (4).
	• Need for small area data means a need for high quality geographic identifiers and new methodology (9).
	• Develop tools for reaching "missing populations" (11).
5.2. Technology	• Look at technology required to support such a system and in particular non-proprietary formats (6).
5.3. Observatory/repository	• Think beyond conventional surveys and consider different modes, e.g. a public health observatory or population health repository, to use existing data (9).
5.4. Emerging issues	• Deal with privacy issues (11).
	• Deal with confidentiality issues (12).
6. Communication	
6.1. Internal communication	• Create an internet-based communication platform which is a professional (secure) site (1).
	• Create an online forum to share experiences about surveys, data, program, and new developments in surveillance (2).
	• Establish library and information exchange forum (12).
6.2. External communication	• Create a web portal for public use (1).
	• Need to use dissemination as a public health strategy (4).
7. Evaluation	
7.1. Demonstration of benefit	• Identify particular issues and areas of greatest surveillance need and pilot surveillance systems to demonstrate its ability to function at all levels (6).
	• Conduct a smaller-scale pilot to demonstrate value and gain buy-in (7).
	• Build business case, with models showing benefits to decision makers and funders (8).

Numbers following items indicate which group(s) generated the response.

#### First, strategy

Both recommendations at the Think Tank Forum and our follow-up review suggest that future consideration should include developing a strategy for surveillance at the regional/local level. The strategy should include marketing and funding strategies, legislation and role of various stakeholders.

#### Second, collaboration

There is a need to be more inclusive in the identification of stakeholders and, in so doing, will have a synergistic effect at all levels. Collaboration does not need to have all opportunities through face-to-face meetings. It can take advantage of the technology available for virtual meetings. Follow up activities have included using technology to facilitate collaboration and promoting recurring opportunities to connect. For example, at the Association of Public Health Epidemiologists in Ontario (APHEO) geographic information systems (GIS) strategic planning session, there were small groups and one of the groups was a virtual e-group. CARRFS has facilitated a number of e-training sessions and an e-symposium in 2012 entitled "From Surveillance to Action: Building Usable Knowledge."

#### Third, information

Information should be made easier to access. This can be done through data sharing agreements. With increased access, there will be increased use of the information, which will lead to increased demand, and in turn will require increased support. Data sharing practices and reporting standards should be made easier. For both data standards and standard questions, there should be a coordinated inventory to increase effectiveness and efficiencies. The Tools and Resources working group of CARRFS is working to build an online inventory to address this need.

#### Fourth, education

Education opportunities should be promoted, and could be virtual. Tool kits could be developed. An educational network should be developed where people would turn to as a first resource. Over the last four years, the CARRFS that was created by the Think Tank Forum has become an important national network of surveillance professionals across Canada [9]. The CARRFS has provided educational and training opportunities through its 2009 and 2012 national symposiums, regional workshops, webinars and e-newsletters.

#### Fifth, novelty

We should encourage people to think outside of the box. We should embrace new technology and take advantage of new opportunities. New challenges will require new ideas and solutions. An example is the use of future electronic surveys that participants can complete unassisted while still ensuring privacy and confidentiality issues. There is user fatigue within the public responding to surveys. This may require education, but more likely, there is a need to demonstrate value added for the participant and how the data will be used to improve their situation or the community in which they live.

#### Sixth, communication

An internet-based communications platform can facilitate sharing of ideas and information among all individuals interested in regional/local surveillance. In 2012 CARRFS set up a virtual community on its website for members and working groups to communicate with one another [9].

#### Seventh, evaluation

Within the evaluation theme, it will be important to define what will be the focus of the demonstration of the benefit, for example, whether it will be based on need, cost, usage, and change.

## Discussion and conclusions

Four years later, it is useful to provide a review and to comment on the findings of the Think Tank Forum based on progress in risk factor surveillance in Canada at the regional/local level. The Think Tank Forum was based on a deliberative process to identify major issues for evidence-informed decision-making. Deliberative processes draw on several forms of evidence to facilitate discussion about how and in what contexts evidence can be used to take action, and can be seen as useful evidence in their own right [18-20]. The Think Tank Forum used a deliberative dialogue, or a face-to-face interaction in which small groups of diverse individuals exchange opinions around a common concern, examine public issues and develop strategies for change [21].

Our deliberative process involved two steps. Step one was collection of data from 108 participants in a one-and-a-half day forum (162 person-days). Because of intellectual interaction of participants with different backgrounds, we believe this has achieved more than a single person can achieve in 162 days. Moreover, the "wisdom of crowds" often creates unexpected insight through the synergy of differing lenses and opinions, thus achieving outcomes beyond that of a single person [22]. Step two was a careful review and interpretation of data through iterations, over four years, by a group of 18 authors of this paper who provided perspectives at federal (4 authors), provincial (6) and local (6) government levels, and university (2). As in all subjective deliberative studies, some limitations such as subjective biases and loss of generalizability may occur.

On reviewing findings of the Think Tank Forum, we think it is helpful to continue to develop conceptual/theoretical frameworks for surveillance at the local level. Initial work includes Capacity Theory which includes the concepts of leadership, will to act, and associated infrastructure components [23,24]. Validation and development of capacity measures for heart health promotion (based on the Singapore Declaration) of both the individual- and organizational-level components and sub-components of the ‘will to act’, ‘infrastructure’ and ‘leadership’ can be a helpful approach to provide a clear vision and define leadership roles [23]. The Framework for Addressing the Global Obesity Epidemic Locally recommends a number of guiding principles for action, including establishing a diverse team of highly motivated and strategically placed individuals, developing a local jurisdictional focus, and building the surveillance system into existing population health initiatives operating in the region [25].

The Think Tank Forum has provided guidance for enhancing capacity in risk factor surveillance at the regional/local level, and has led to the creation of the Canadian Alliance for Regional Risk Factor Surveillance (CARRFS). It is beneficial to relate CARRFS activities back to the recommendations of the Think Tank Forum which created CARRFS in the first place. CARRFS members and working groups have undertaken a number of activities stemming from these recommendations during the intervening four years. Significant progress has been made in the areas of collaboration, coordination, information, and education. This progress builds an excellent foundation on which to move forward in the areas of strategy, novelty and evaluation. This report and recommendations of the Think Tank Forum in light of progress in the last four years are useful for continuing to build surveillance capacity, both in Canada and globally.

## Appendix

Examples of risk factor surveillance activities at the provincial/territorial and regional/local area level in Canada to collect additional data from local surveys and administrative databases to provide sub-national estimates.

### Alberta

**Alberta Ministry of Health survey program** – The objectives of this provincial program are to address gaps in the national health surveys and to be responsive to provincial needs. It includes surveys in areas of depression, wellness, immunization, and West Nile Virus seroprevalence. The provincial level program fills gaps from the national, and is also more flexible and easier to change direction.

**Alberta Cancer Board survey program** – This survey program is run by the Alberta Cancer Board which is primarily focused on cancer risk factors.

### British Columbia

**British Columbia Health and Wellness Survey (BC-HWS) **– This is an initiative of the Provincial Health Services Authorities (PHSA) in collaboration with the regional health authorities. The survey aims at understanding chronic conditions and health-related lifestyle patterns of British Columbians in 26 local health areas (LHAs) and communities in the province. Survey variables include chronic conditions (diabetes, hypertension, and body mass index), lifestyle risk factors (physical activity, and tobacco, alcohol and fruit and vegetable consumption), and social factors (food access and security).

### Saskatchewan

**Saskatchewan local area surveillance** – In Saskatchewan, the asthma control initiative provides collaborative local area surveillance that involves multiple agencies [26]. The health district asthma survey revealed that local environmental factors such as tobacco smoke, home mould and dampness were significant risk factors associated with asthma in children [27,28].

**Saskatoon Health Region supplementary health surveys** – In Saskatoon Health Region, supplementary health surveys (including self reported disease rates, health behaviours, and risk factors) are conducted periodically for purposes of public health surveillance, research and needs assessment, and reported to the community and to decision makers via health status reports and in the peer reviewed literature [5].

### Manitoba

**Manitoba Partners in Planning for Healthy Living** – This collaborative consists of 23 member organizations including all Regional Health Authorities (including CancerCare Manitoba), four Provincial Ministries, several non-government organizations and other groups who share a common goal – the prevention of chronic diseases. The partners work together to build a Manitoba risk factor surveillance system while recognizing the uniqueness within each Manitoba community. Some of the activities include local risk factor surveillance, knowledge exchange, program and policy development, implementation, evaluation, and academic research. A Manitoba youth health survey has been implemented to provide local risk factor data to each school, school division, community and region, and rolled up to provide a provincial YHS report. Adult Health Surveys are being developed for use in Manitoba communities.

### Ontario

**Rapid Risk Factor Surveillance System (RRFSS)** – A pilot project of monthly surveys was conducted in the Durham Health Region in 1999, because some local health regions felt the need to have a rapid and flexible information system to supplement data from national health surveys [4]. As a result of the successful pilot project that was supported at the national, provincial and local levels, RRFSS was set up in Ontario and has been in operation ever since [6].

**Ontario Student Drug Use and Health Survey (OSDUHS)** – A population survey of Ontario students in grades 7 to 12 since 1977, OSDUHS is the longest ongoing school survey in Canada. The survey is conducted across the province every two years for identifying epidemiological trends in student tobacco and drug use, mental health, physical activity and other risk behaviours [29].

**Ontario School Health Environment Survey (SHES)** – The purpose of SHES is to assess factors in the school environment that contribute to healthy eating and physical activity among children and youth. Questions were asked about specific aspects of the school environment pertaining to the promotion of healthy eating and physical activity. Results from SHES help schools and school boards assess how supportive they are towards healthy eating and physical activity.

**The School Health Action, Planning and Evaluation System (SHAPES)** – SHAPES collects data from local elementary and high schools on topics such as smoking, eating and physical activity. Survey data is used to generate profiles to help schools, public health, and communities to take action to improve the health of young people [30]^1^.

**Perth District Health Unit (PDHU) Parent and Adult surveys** – The PDHU collected local data through Parent and Adult surveys. The purpose of the surveys is to learn more about behaviours, knowledge and opinions with regard to health of children and adults, and to promote familiarity of Perth County residents with locally offered programs in the community [31,32].

**The Wellness Clinic for Tots** – The objectives of this project are to increase early identification of children at risk for growth and developmental delays, health and nutrition concerns; increase timely referral of identified concerns; reduce risk of obesity among preschoolers; educate parents on healthy lifestyles and child development, nutrition and local supports; and increase outreach to cultural communities [33].

### Nova Scotia

**Nova Scotia Health Survey** – The Nova Scotia Health Survey is a follow up to the previous Nova Scotia Heart Health Survey [34].

**District Health Authorities surveys** – Colchester East Hants Health Authority (CEHHA) conducted a survey of youths in junior and senior high schools to inform Primary Health Care planning at the district level. Guysborough Antigonish Strait Health Authority (GASHA) and Cape Breton District Health Authority (CBDHA) conducted the "Understanding Our Health" survey using selected questions from the CCHS in order to provide statistically valid results for each of the Community Health Board (CHB) areas that comprise a District.

## Competing interests

The authors declare that they have no competing interests.

## Authors’ contributions

BC conceived the study. All authors contributed to data collection, analysis and interpretation. All authors read and approved the final manuscript.
